# Association between physical activity and the prevalence of tumorigenic bacteria in the gut microbiota of Japanese adults: a cross-sectional study

**DOI:** 10.1038/s41598-023-47442-9

**Published:** 2023-11-27

**Authors:** Chiharu Iwasaka, Yuka Ninomiya, Takashi Nakagata, Hinako Nanri, Daiki Watanabe, Harumi Ohno, Kumpei Tanisawa, Kana Konishi, Haruka Murakami, Yuta Tsunematsu, Michio Sato, Kenji Watanabe, Motohiko Miyachi

**Affiliations:** 1 Department of Physical Activity Research, National Institutes of Biomedical Innovation, Health and Nutrition, Tokyo, Japan; 2 Laboratory of Gut Microbiome for Health, Microbial Research Center for Health and Medicine, National Institutes of Biomedical Innovation, Health and Nutrition, Osaka, Japan; 3 Faculty of Sport Sciences, Waseda University, Saitama, 359-1192 Japan; 4 Department of Nutrition, Kiryu University, Gunma, Japan; 5 Faculty of Food and Nutritional Sciences, Toyo University, Gunma, Japan; 6 Faculty of Sport and Health Science, Ritsumeikan University, Shiga, Japan; 7 Department of Pharmaceutical Sciences, University of Shizuoka, Shizuoka, Japan

**Keywords:** Microbiology, Health care, Disease prevention, Public health, Cancer prevention

## Abstract

*Escherichia coli* harboring polyketide synthase (*pks*^+^ *E. coli*) has been suggested to contribute to colorectal cancer development. Physical activity is strongly associated with lower colorectal cancer risks, but its effects on *pks*^+^ *E. coli* remain unclear. The aim of this study was to investigate the association between *pks*^+^ *E. coli* prevalence and physical activity. A cross-sectional study was conducted on 222 Japanese adults (27–79-years-old, 73.9% female). Triaxial accelerometers were used to measure light-intensity physical activity, moderate-to-vigorous intensity physical activity, the physical activity level, step-count, and time spent inactive. Fecal samples collected from participants were used to determine the prevalence of *pks*^+^ *E. coli*. Multivariate logistic regression analysis and restricted cubic spline curves were used to examine the association between *pks*^+^ *E. coli* prevalence and physical activity. The prevalence of *pks*^+^ *E. coli* was 26.6% (59/222 participants). The adjusted odds ratios (ORs) and 95% confidence intervals (CIs) for the highest tertile with reference to the lowest tertile of physical activity variables were as follows: light-intensity physical activity (OR 0.63; 95% CI 0.26–1.5), moderate-to-vigorous intensity physical activity (OR 0.85; 95% CI 0.39–1.87), physical activity level (OR 0.69; 95% CI 0.32–1.51), step-count (OR 0.92; 95% CI 0.42–2.00) and time spent inactive (OR 1.30; 95% CI 0.58–2.93). No significant dose–response relationship was found between all physical activity variables and *pks*^+^ *E. coli* prevalence. Our findings did not suggest that physical activity has beneficial effects on the prevalence of *pks*^+^ *E. coli*. Longitudinal studies targeting a large population are needed to clarify this association.

## Introduction

Colorectal cancer (CRC) is the third most common malignancy worldwide and the second most common cause of cancer-related deaths^1^. In the future, it is estimated that the global burden of CRC will increase by 60%, resulting in more than 2.2 million new patients and 1.1 million deaths by 2030^2^. Modifiable risk factors, including alcohol intake, smoking, obesity, poor diets, and physical inactivity, are widely recognized as known risk factors for CRC^3, 4^. In addition to these, gut microbiota has emerged in recent years as an important risk factor for CRC and is receiving increasing attention^5^.

In recent years, it has been suggested that *Escherichia coli* of the B2 phylogenetic group, which has a genomic island called polyketide synthetase (*pks*^+^ *E. coli*), might be involved in the development of CRC^6–12^. *pks*^+^ *E. coli* encodes the genotoxin colibactin, which induces DNA damage, cell cycle arrest, mutations, and chromosomal instability in eukaryotic cells^7, 8, 13^. Indeed, the results of a previous meta-analysis demonstrated a CRC odds ratio (OR) of 2.3 for *pks*^+^ *E. coli* carriers compared to non-carriers^11^. In addition, since small molecule inhibitors targeting colibactin production have been reported to prevent tumorigenesis in mouse models^14^, strategies to reduce the prevalence of *pks*^+^ *E. coli* could lead to the prevention of CRC.

Physical activity (PA) serves as a crucial preventive measure against colorectal cancer (CRC)^15^. A meta-analysis has shown that high levels of PA are associated with a 23% reduced risk of CRC compared to low PA levels^16^. Furthermore, recent findings indicate that the gut microbiota may play a pivotal role in mediating the relationship between PA and the reduced risk of CRC development^17–19^. A key factor in understanding the mechanism underlying the preventive effect of PA against CRC is short-chain fatty acids (SCFAs), metabolites produced by specific gut microbiota^20^. SCFAs are generated through the fermentation of dietary fiber by the gut microbiota^21^. They serve as an energy source for intestinal epithelial cells and have anti-inflammatory, pH-regulating, gut motility-enhancing, barrier function-enhancing, and antineoplastic properties^20, 21^. Under colonic conditions, SCFAs notably inhibit the growth of pathosymbiont *E. coli* and suppress its virulence genes, including the genotoxicity-associated *pks* gene cluster^22^. A recent meta-analysis revealed that lower fecal SCFA concentrations correlate with a higher risk and incidence of CRC^23^. PA has been associated with an increase in SCFA-producing bacteria and elevate fecal SCFA concentrations^24, 25^. Thus, regular PA might deter the colonization and proliferation of *pks*^+^ *E. coli* by enhancing SCFA production.

Although these previous studies provide important insights into reducing the prevalence of *pks*^+^ *E. coli*, several knowledge gaps exist. First, to our knowledge, no studies have examined the association between PA and *pks*^+^ *E. coli*, and thus, it remains unclear whether they are related to each other. Second, some studies suggest that excessive PA could have an adverse effect on gut microbiota^26, 27^. Specifically, prolonged or high-intensity exercise has been reported to decrease the diversity of gut microbiota and increase inflammatory bacteria, while the optimal amount or intensity of PA remains unknown^27^. Therefore, the intensity and dose–response relationship of PA against *pks*^+^ *E. coli* should also be evaluated. Furthermore, the beneficial effects of PA on *pks*^+^ *E. coli* could be partially mediated by an increase in SCFA levels, but this association is also not well understood. To address these gaps, we investigated the association between objectively measured PA using a tri-axial accelerometer and the prevalence of *pks*^+^ *E. coli* in Japanese individuals 20 years of age or older. We hypothesized that PA is inversely associated with the prevalence of *pks*^+^ *E. coli* and that this association is partially mediated by SCFAs.

## Results

Table 1 shows the demographic characteristics of the groups with and without *pks*^+^ *E. coli*. Of the 222 participants, 59 were in the *pks*^+^ *E. coli* group (26.6%) and 163 were in the *pks*^*−*^* E. coli* group. The *pks*^+^ *E. coli* group was characterized by a significantly lower percentage of females, shorter light-intensity PA (LPA) time, longer inactivity time, lower green tea intake, and a lower percentage of alcohol-drinkers than the *pks*^*−*^* E. coli* group (*P* < 0.05). The demographic characteristics based on the tertiles of each PA variable (LPA, moderate-to-vigorous-intensity PA [MVPA], inactivity time, PA level [PAL], and step-count) are shown in Supplementary Tables S1–S5.

**Table 1:** Demographic characteristics of *pks*^*−*^* E. coli* and *pks*^+^ *E. coli*.

Characteristics	Overall, n = 222	*pks*^*−*^* E. coli*, n = 163	*pks*^+^ *E. coli*, n = 59	*P*-value
Age, y	58.7 (12.3)	59.1 (12.2)	57.7 (12.7)	0.454
Female, n (%)	164 (73.9%)	128 (78.5%)	36 (61.0%)	0.009
Height, cm	160.3 (8.1)	159.8 (7.8)	161.7 (8.8)	0.139
Weight, kg	57.8 (9.2)	57.5 (9.2)	58.7 (9.3)	0.380
BMI, kg/m^2^	22.4 (2.7)	22.5 (2.8)	22.4 (2.6)	0.858
Family history of cancer, n (%)	125 (56.3%)	97 (59.5%)	28 (47.5%)	0.110
LPA, min/day	354 (99)	363 (99)	328 (98)	0.023
MVPA, min/day	64 (29)	64 (29)	64 (30)	0.962
Inactivity, min/day	1023 (106)	1,013 (105)	1,048 (105)	0.033
PAL	1.75 (0.15)	1.76 (0.15)	1.73 (0.16)	0.150
Step-count, steps/day	9634 (3266)	9,653 (3400)	9582 (2887)	0.876
Energy intake, kcal/day	1728 (450)	1712 (410)	1774 (547)	0.425
Green tea intake, g/1000 kcal/day	136 (136)	151 (143)	94 (103)	0.001
Alcohol drinker, n (%)	115 (51.8%)	92 (56.4%)	23 (39.0%)	0.021
Smoking, n (%)				0.215
Current	8 (3.6%)	4 (2.5%)	4 (6.8%)	
Former	50 (22.5%)	35 (21.5%)	15 (25.4%)	
Never	164 (73.9%)	124 (76.1%)	40 (67.8%)	

*pks*^+^ *E. coli*, polyketide synthase *Escherichia coli* positive; *pks*^*−*^* E. coli*, polyketide synthase *Escherichia coli* negative; BMI, body mass index; LPA, light-intensity physical activity; MVPA, moderate-to-vigorous physical activity; PAL, physical activity level. Continuous: mean (SD), tested with a t-test; categorical: n (%), tested with a chi-square test.

Table 2 shows the prevalence of *pks*^+^ *E. coli* in each PA variables tertile and the results of the logistic regression analysis. In Model 1, only LPA observed a significant inverse association with the prevalence of *pks*^+^ *E. coli* (*P* for trend = 0.027), but significance was lost in Model 2 adjusted for age and sex (*P* for trend = 0.241). Fully adjusted ORs and CIs for T3, with T1 as a reference, were as follows: LPA (Model 3: OR 0.63; 95% CI 0.26–1.52, *P* for trend = 0.297), MVPA (Model 3: OR 0.85; 95% CI 0.39–1.87, *P* for trend = 0.694), Inactivity (Model 3: OR 1.30; 95% CI 0.58–2.93, *P* for trend = 0.460), PAL (Model 3: OR 0.69; 95% CI 0.32–1.51, *P* for trend = 0.345), and step-count (Model 3: OR 0.92; 95% CI 0.42–2.00, *P* for trend = 0.847). No significant associations were observed between the prevalence of *pks*^+^ *E. coli* and all PA variables (*P* for trend > 0.05). Post-hoc statistical power calculations revealed low power for all PA variables, as follows: 0.38 for LPA, 0.08 for MVPA, 0.17 for inactivity time, 0.27 for PAL, and 0.06 for the step-count.

**Table 2:** Adjusted odds ratios and 95% confidence intervals for each PA variable for the prevalence of *pks*^+^ *E. coli*.

Exposure variables	Median (min–max)	n	*pks*^+^ *E. coli*, n	Prevalence, %	Model 1			Model 2			Model 3		
					OR	95% CI		OR	95% CI		OR	95% CI	
						Lower	Upper		Lower	Upper		Lower	Upper
LPA, min/day													
T1	240 (146–302)	74	26	35.1	Reference			Reference			Reference		
T2	351 (305–398)	74	19	25.7	0.64	0.32	1.29	0.79	0.37	1.69	0.73	0.33	1.61
T3	458 (399–584)	74	14	18.9	0.43	0.20	0.91	0.60	0.26	1.41	0.63	0.26	1.52
*P* for trend					0.027			0.241			0.297		
MVPA, min/day													
T1	37 (16–50)	74	21	28.4	Reference			Reference			Reference		
T2	60 (50–69)	74	19	25.7	0.87	0.42	1.80	0.83	0.40	1.74	0.94	0.43	2.04
T3	89 (70–215)	74	19	25.7	0.87	0.42	1.80	0.78	0.37	1.66	0.85	0.39	1.87
*P* for trend					0.710			0.522			0.694		
Inactivity, min/day													
T1	920 (777–965)	74	18	24.3	Reference			Reference			Reference		
T2	1024 (970–1069)	74	14	18.9	0.73	0.33	1.60	0.63	0.28	1.41	0.53	0.23	1.24
T3	1142 (1071–1241)	74	27	36.5	1.79	0.88	3.64	1.31	0.60	2.85	1.30	0.58	2.93
*P* for trend					0.096			0.442			0.460		
PAL													
T1	1.60 (1.39–1.67)	74	26	35.1	Reference			Reference			Reference		
T2	1.74 (1.68–1.82)	74	16	21.6	0.51	0.25	1.06	0.61	0.29	1.30	0.57	0.26	1.25
T3	1.90 (1.82–2.34)	74	17	23.0	0.55	0.27	1.13	0.66	0.31	1.39	0.69	0.32	1.51
*P* for trend					0.096			0.269			0.345		
Step-count, steps/day													
T1	6810 (3712–8212)	74	21	28.4	Reference			Reference			Reference		
T2	9206 (8269–10,558)	74	16	21.6	0.70	0.33	1.47	0.65	0.30	1.40	0.66	0.29	1.49
T3	12,399 (10,570–29,432)	74	22	29.7	1.07	0.53	2.17	0.88	0.42	1.85	0.92	0.42	2.00
*P* for trend					0.852			0.737			0.847		

PA, physical activity; *pks*^+^ *E. coli*, polyketide synthase Escherichia coli positive; OR, odds ratio; CI, confidence interval; LPA, light-intensity physical activity; MVPA, moderate-to-vigorous physical activity; PAL, physical activity level; T, tertile.

Model 1: crude model.

Model 2: adjusted for age and sex.

Model 3: model 2 + body mass index, family history of cancer, smoking, alcohol drinking, energy intake, and green tea intake.

We observed no significant interactions between sex or the age group (60 + vs. < 60 years) and PA variables in relation to the prevalence of *pks*^+^ *E. coli*. For sex interactions, *P*-values were as follows: LPA, 0.618; MVPA, 0.176; inactivity, 0.393; PAL, 0.810; step-count, 0.416. For age group interactions, *P*-values were as follows: LPA, 0.178; MVPA, 0.539; inactivity, 0.178; PAL, 0.420; step-count, 0.639.

**Figure 1:**
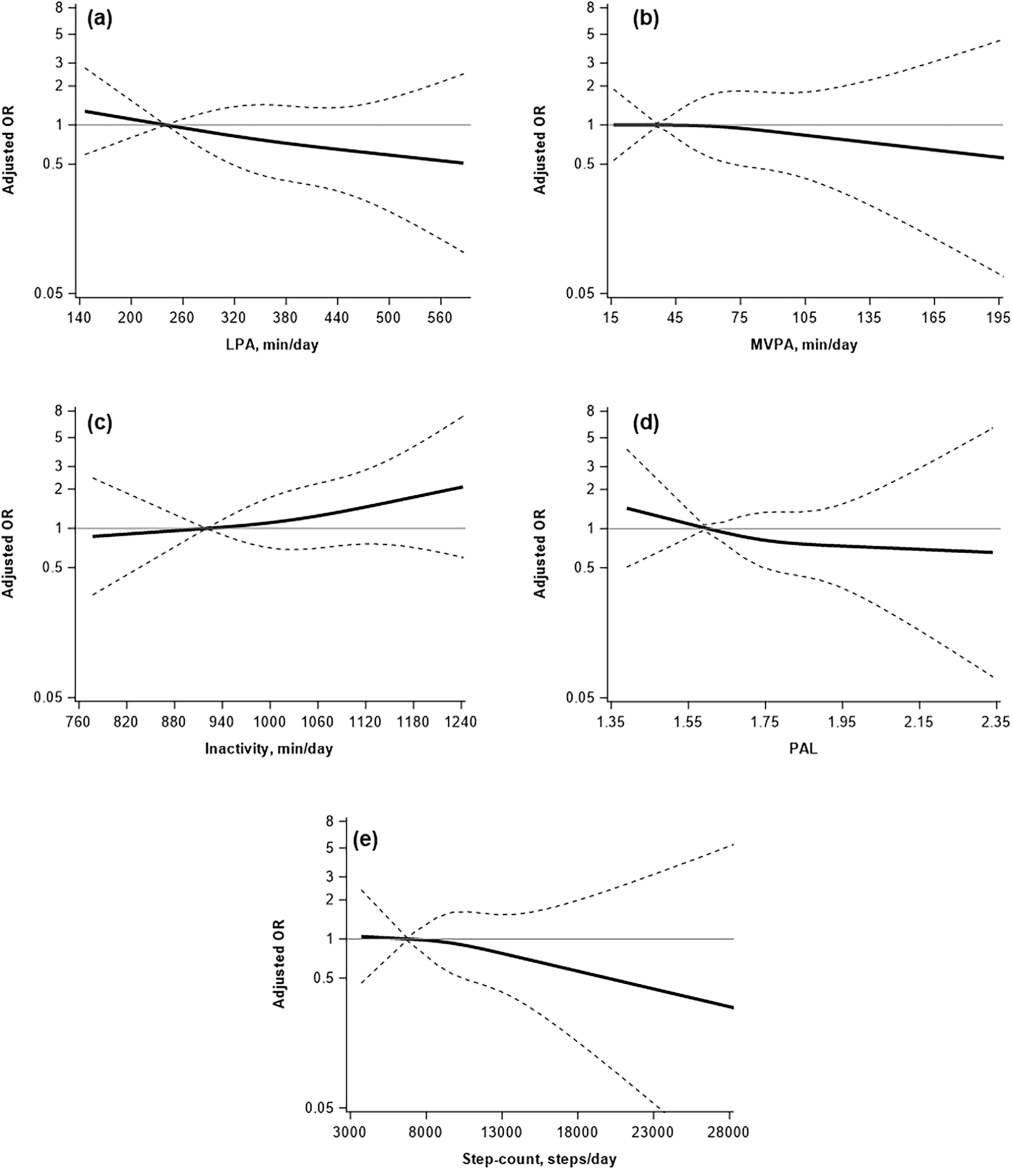
Restricted cubic spline curves showing the dose–response relationship between the prevalence of *pks*^+^ *Escherichia coli* and each physical activity variable. Graphs depict (**a**) Light intensity physical activity (LPA), (**b**) moderate-to-vigorous physical activity (MVPA), (**c**) time spent inactive, (**d**) physical activity level (PAL), (**e**) step-count. Solid lines represent odds ratios and dashed lines represent 95% confidence intervals. The Y-axis is shown on the logarithmic axis. All dose–response relationships were adjusted for age, sex, body mass index, drinking, smoking, a family history of cancer, energy intake, and green tea intake.

Figure 1 shows the dose–response relationship of each PA variable with respect to the prevalence of *pks*^+^ *E. coli* using a cubic spline curve. The 95% CIs for all PA variables were wide, and no significant dose–response relationships were observed (*P* > 0.05). In the spline model, the interaction between neither the sex nor the age group and PA variables was significant (Supplementary Figs. S1 and S2, *P* for both interactions > 0.05). Supplementary Tables S6–S10 present the results of the mediation analysis using fecal SCFAs as a mediating factor. No mediation effects of SCFAs were observed for all PA variables (*P* > 0.05).

## Discussion

The aim of this study was to examine the association between PA and the prevalence of *pks*^+^ *E. coli*. Contrary to our hypothesis, there was no clear association between the prevalence of *pks*^+^ *E. coli* and the amount or intensity of PA. No significant dose–response relationship was observed either. The results of this study did not support our hypothesis that PA promotion is inversely associated with the prevalence of *pks*^+^ *E. coli*.

In the fully adjusted model, the association between PA variables and the prevalence of *pks* + *E. coli* was not statistically significant. Nonetheless, these results warrant careful interpretation. In this study, the ORs for the highest tertile, compared to the lowest tertile of PA variables, ranged from 0.63 to 0.92 (with 1.3 for inactivity). The restricted cubic spline curves also demonstrated a trend of decreasing odds ratios (or increasing for inactivity) with each increment in PA variables. However, these trends did not attain statistical significance, and they were accompanied by a wide 95% CI. Based on the PAL results from our study, we estimated that a sample size of 1009 participants (power = 0.8, α = 0.05, prevalence of *pks*^+^ *E. coli* in T1 = 0.35, and OR per one category increase based on the PAL = 0.83) would be needed to detect a significant difference. The inability of our study to identify a significant association between PA and *pks*^+^ *E. coli* might be attributed to our limited sample size. Larger cohort studies in the future could offer more definitive insights into this association.

On the other hand, we observed a significant inverse association between LPA and *pks*^+^ *E. coli* prevalence in unadjusted models. This inverse correlation may have been contributed by sex as a confounding factor. We performed additional analyses to include age and sex separately in the logistic regression model and confirmed that the association between LPA and *pks*^+^ *E. coli* prevalence is lost when adjusting for sex only (sex-only adjustment model, *P* for trend = 0.22; age-only adjustment model, *P* for trend = 0.036). To clarify the influence of sex, we also examined the interaction between sex and PA, but did not observe significant sex differences in the association between *pks*^+^ *E. coli* prevalence and PA. However, note that 74% of the participants in this study were female. Our previous reports indicate that females have a lower prevalence of *pks*^+^ *E. coli* than males^28^. In addition, previous studies suggest that males may be more likely than females to benefit from PA-induced reductions in CRC risk due to sex hormones^29^. Although no significant interaction between PA and sex was observed in this study, the high proportion of female participants may have been one factor that attenuated the association between PA and *pks*^+^ *E. coli* prevalence.

In addition, many of the participants in this study may have engaged in high levels of PA. According to data published by the Japanese Ministry of Health, Labour and Welfare, the average step-count per day for Japanese adults is 6793 for males and 5832 for females^30^, whereas the average step-count per day for the participants in this study was 10,634 for males and 9281 for females (data not shown). Therefore, it is possible that the participants in this study likely engaged in more PA than the general Japanese population and that association with the prevalence of *pks*^+^ *E. coli* in those with low PA may have been underestimated.

We initially postulated that SCFAs might partially mediate the relationship between PA and *pks*^*+*^* E. coli*. However, our mediation analysis results did not support the anticipated association among PA, SCFAs, and *pks*^*+*^* E. coli*. Notably, existing literature does not unanimously endorse the beneficial effects of SCFAs. In one particular study, elevated fecal SCFA levels in the general adult population were associated with an increased occurrence of gut dysbiosis, increased gut permeability, cardiometabolic risk factors, and obesity^31^. In a study focusing on community-dwelling older adults with insomnia, those with lower PALs, as gauged by accelerometers, exhibited higher fecal SCFA concentrations^32^. Another recent investigation revealed an inverse relationship between MVPA, as measured by accelerometers, and fecal SCFA concentrations^25^. One possible explanation for these findings is the limited absorption of SCFAs in the gut, which results in their excretion in feces^33^. However, it is worth noting that the utility of SCFAs as a biomarker for tumorigenesis prevention has been questioned by some researchers^34^. Given these diverse findings, it is evident that the relationship among PA, SCFAs, and *pks* + *E. coli* is complex and warrants further investigation. Future studies should aim to provide a clearer understanding of the intricate interplay among these factors.

Although some studies have suggested the negative effect of excess PA on gut microbiota^26, 27^, our results did not seem to indicate that a higher PA level was associated with the increased induction of *pks*^+^ *E. coli*. The findings that gastrointestinal disorders and inflammation are promoted in the gut due to intense exercise have depended primarily on findings from endurance athletes^27, 35^. In addition, the participants in this study were adults of the general population, and VPA as a percentage of total PA was negligible (0.3% of total PA in this study). Therefore, PA at the level at which the general public is engaged is not considered harmful enough to increase the prevalence of *pks*^+^ *E. coli*.

We previously reported that green tea intake or stool patterns are associated with *pks*^+^ *E. coli* prevalence in the Japanese population^28, 36^. The results of this study may suggest that the influence of PA on the prevalence of *pks*^+^ *E. coli* is weaker than that of dietary and stool factors. The development of CRC is not solely attributable to the presence of *pks*^+^ *E. coli*^12^. Numerous factors contribute to the risk of CRC, including diet, genetics, lifestyle^4^. Previous studies have indicated an inverse association between PA and CRC incidence^16^, suggesting that PA might influence CRC risk through various mechanisms. Regulation of inflammation, apoptosis, growth factor axis, immunity, and epigenetic factors have been reported as underlying mechanisms association between PA and lower CRC risk, although they are not fully understood^37^. Our study only examined the association between PA and *pks*^+^ *E. coli* prevalence, which is only one of the risk factors for CRC. Based on our findings alone, it would be premature to conclude that PA does not influence the prevalence of *pks*^+^ *E. coli*. Our findings are observational and preliminary, requiring cautious interpretation and further research.

This study had several limitations that should be mentioned. Firstly, due to its cross-sectional design, we cannot infer causality from the observed associations. Secondly, the sample size poses a concern. As highlighted in the discussion, our study may lack the statistical power to detect subtle associations between PA and the prevalence of *pks*^+^ *E. coli*. Third, the sex distribution was skewed, with female participants comprising 74% of the cohort. A stratified analysis by sex might shed light on the potential influence of sex on the association. However, this study had insufficient statistical power to perform a stratified analysis by sex. Future research involving larger and more balanced samples will be instrumental for clarifying this association. Lastly, our study did not employ random selection of participants from the city, indicating a potential selection bias.

In conclusion, *pks*^+^ *E. coli* is a new risk factor for the development of CRC, and the search for modifiable environmental factors to establish primary prevention strategies is essential. The results of this study found no clear association between PA and *pks*^+^ *E. coli* and it remains unclear whether PA reduces the prevalence of *pks*^+^ *E. coli*. Longitudinal and interventional studies based on larger populations are needed to clarify the association between PA and *pks*^+^ *E. coli* prevalence.

## Methods

### Study design and procedure

This cross-sectional study utilized the same cross-sectional dataset from the Nutrition and Exercise Intervention study (NEXIS) as our previous studies, which reported associations between green tea intake or stool patterns and the prevalence of *pks*^+^ *E. coli* in the Japanese population^28, 36^. Briefly, the NEXIS is a longitudinal cohort initiated in 2012 with the aim of evaluating the association between lifestyle and health markers such as dietary intake and physical activity^28, 36^. Of the 750 general Japanese adults who participated in the NEXIS, 259 individuals, ranging in age from 27 to 79 years who were living in the Tokyo metropolitan area in Japan, participated in a stool sampling survey that included measurement of the prevalence of *pks*^+^ *E. coli*^28, 36^. Therefore, the sample size for this study was not determined based on specific statistical analyses but rather on the availability of data.

Participants were mailed a dietary and lifestyle questionnaire and a fecal sampling and storage kit (TechnoSuruga Laboratory Co., Ltd, Shizuoka, Japan)^38^ prior to the face-to-face survey. The self-administered questionnaire included medical history, smoking status, dietary habits, and stool condition. Dietary intake was assessed using a validated self-administered diet history questionnaire consisting of 58 items^39^. The stool condition was assessed using a validated card tool that questions the volume, form, color, and odor of the stool^40^. Participants were instructed to collect a fecal mass of approximately 2 cm in diameter (approximately 3 g) at home using a stool collection kit. The fecal samples collected were sealed in a special container and stored at − 20 °C. Participants brought their fecal storage kits and questionnaires within 5 days of fecal sampling and participated in a face-to-face survey that included anthropometric measurements, physical fitness tests, blood tests, and vascular function tests. Incomplete questionnaires were verified via interview by survey staff (registered dietitians and nurses). Frozen stool samples were transported in a refrigerated truck to the University of Shizuoka for the detection of *pks*^+^ *E. coli*. Approximately 7 mm of the cryopreserved fecal samples were used for DNA extraction. This survey was conducted between September 2015 and December 2017.

This study received approval from the Research Ethics Review Committee of the National Institutes of Biomedical Innovation, Health and Nutrition (No. Kenei 3-10 and Kenei 102-04). The study’s procedures and associated risks were thoroughly explained to all subjects, and written informed consent was acquired from every participant. The research was conducted adhering to the principles of the Declaration of Helsinki. Out of the 259 participants, those with histories of conditions such as cancer (n = 13), inflammatory bowel disease (n = 2), irritable bowel syndrome (n = 1), diabetes (n = 13), renal failure (n = 1), cardiovascular disease (n = 6), and those with missing accelerometer data (n = 1) were excluded from the study. As a result, 222 participants were incorporated into the final analysis.

### Determination of *pks*^+^*E. coli* by polymerase chain reaction (PCR)

PCR was performed using SapphireAmp Fast PCR Master Mix (Takara Bio Inc., Shiga, Japan) according to the manufacturer’s protocol. The primer sets used were as follows: *clbB* forward primer, 5′-tgttccgttttgtgtggtttcagcg-3′; reverse primer, 5′-gtgcgctgaccattgaagatttccg-3′; *clbJ* forward primer, 5′-tggcctgtattgaaagagcaccgtt-3′; reverse primer, 5′-aatgggaacggttgatgacgatgct-3′; *clbQ* forward primer, 5′-ctgtgtcttacgatggtggatgccg-3′; reverse primer, 5′-gcattaccagattgtcagcatcgcc-3′. We defined a *pks*^+^ *E. coli* carrier when *clbB*, *clbJ*, or *clbQ* was detected in the feces using these primers^28, 36^. The minimum detection level of *clb* genes by PCR was estimated at 10 ng/mL as a DNA template^36^.

### Fecal SCFA measurement

The data set used in this study included fecal SCFA measured in 160 of 222 individuals for another study of ours previously reported on the association between stool patterns and *pks*^+^ *E. coli* and fecal SCFA^36^. Therefore, this SCFA value was used as a potential mediating variable in the association between the PA variable and *pks*^+^ *E. coli*. Briefly, to measure the fecal SCFA content, 5–10 mg of feces from each of the selected participants was mixed with 90 μL of Milli-Q water and 10 μL of 2 mM internal standard containing acetic acid, butyric acid, and crotonic acid, and the mixture was allowed to sit for 5 min. The mixture was then homogenized with 50 μL of 36% HCl and 200 μL of 97% diethyl ether. This homogenized mixture was centrifuged at 3000 rpm for 10 min at room temperature. 80 μL of the supernatant organic layer was carefully transferred to a new glass vial and combined with 16 μL of *N*-tert-butyldimethylsilyl-*N*-methyltrifluoroacetamide for derivatization. The vials were capped immediately with an electronic crimper (Agilent) and incubated for 20 min in an 80 °C water bath, then left at room temperature in the dark for 48 h for complete derivatization. The derivatized samples were analyzed using a GC-MS-TQ8040 gas chromatograph mass spectrometer (Shimadzu Corporation, Kyoto, Japan), with injection performed using an AOC-20i autoinjector (Shimadzu Corporation, Kyoto, Japan). The capillary column was a BPX5 column (0.25 mm × 30 m × 0.25 μm; Shimadzu GLC), with pure helium gas used as the carrier gas at a flow rate of 1.2 mL min^−1^. The head pressure was 72.8 kPa with a split ratio of 30:1. The injection port and interface temperatures were maintained at 230 °C and 260 °C, respectively. In this study, the total SCFA content (mean: 95.5 mol/g; range: 7.99–204.5) was used for the mediation analysis.

### Objective evaluation of PA parameters

PA was monitored using a triaxial accelerometer (Actimarker EW4800, Panasonic Electric Works, Osaka, Japan; dimensions, 74 × 33 × 13 mm; weight, 24 g), which uses an algorithm that has been validated using a metabolic chamber and the doubly labeled water method^41, 42^. Participants were instructed to wear the accelerometer on their waist while awake for 28 days, with the exception of bedtime, showering/bathing, and water activities^43^. Valid wearing days required a total of ≥ 7 days of accelerometer data with at least 10 h of accelerometer wear per day. If the number of valid days was not met, participants were asked to wear the accelerometer again. PA time per day was calculated by summing the PA time observed during the measurement period and dividing it by the number of valid days. The 24 h average metabolic equivalent (MET) was obtained from the triaxial accelerometers, and the total energy expenditure (TEE) was calculated based on the following equation, considering diet-induced thermogenesis to be 10% of the TEE: TEE (kcal/day) = (predicted basal metabolic rate [BMR] × 24 h average METs)/0.9^44^. Then, we used the mean value of the included data as the representative value of the individual for the analysis. The PA level (PAL) was calculated by dividing TEE by BMR. The PA parameters used in the analysis were as follows: light-intensity PA (LPA, 1.5–2.9 METs/day), moderate-to-vigorous-intensity PA (MVPA, ≥ 3 METs/day), PAL, and step-count per day. Time spent inactivity was defined as the sum of sedentary (< 1.5 METs) and non-wearing time and calculated as 1440 − (LPA + MVPA) based on previous studies^45^. These PA intensity categories have been commonly used in previous studies using the same device^43, 46, 47^. In our study, we used an accelerometer that does not have the capability of distinguishing among the sedentary time, sleep time, and non-wearing time (such as during bathing or swimming). Therefore, our analysis treated these periods as the combined inactive time spent.

### Statistical analysis

Participant characteristics were expressed as arithmetic means and standard deviations for continuous variables, and as the number of individuals and percentages for categorical variables. Differences in characteristics between groups with and without *pks*^+^ *E. coli* were compared by performing a *t*-test for continuous variables and by performing a chi-square test for categorical variables. Each PA variable (LPA, MVPA, inactivity, PAL, and step-count) was categorized into tertiles; linear regression analysis was used for continuous variables, and Mantel–Haenszel tests were used for categorical variables to examine linear trends among the tertiles. Multivariate logistic regression analysis was used to examine the association between each PA variable and *pks*^+^ *E. coli* prevalence. We calculated the odds ratios (ORs) and 95% confidence intervals (CIs) for prevalence of *pks*^+^ *E. coli* for each tertile, using the lowest tertile as a reference. Based on previous studies^28^, the following variables were used as covariates; age (continuous; years), sex (category; male or female), BMI (continuous; kg/m^2^), family history of cancer (category; yes or no), alcohol consumption (category; yes or no), smoking (category; current, former, or never), energy intake (continuous; kcal/day), and green tea consumption (continuous; g/1000 kcal/day). Liner trend tests were performed by changing the tertile categorical variables to ordinal scales. We tested for potential interaction effects by sex and age groups on the association between the PA variables and *pks* + *E. coli*. Interaction terms (sex × PA variables and age group [60 + vs. < 60] × PA variables) were added to the multivariate logistic analysis model.

In addition, the spline effect statement of the logistic regression model was used to evaluate the dose–response relationship of each PA variable on the prevalence of *pks*^+^ *E. coli*. The number of knots was set at three (knots located at the 10th, 50th, and 90th percentiles)^48^. The reference value was set to the median of the lowest tertile. The y-axis of the restricted cubic spline curve was expressed on the logarithmic axis. Similarly, in the spline model, we examined the interactions between sex or age group (60 + vs. < 60) and PA variables in relation to the prevalence of *pks*^+^ *E. coli*. Furthermore, a mediation analysis with SCFAs as the mediating variables was conducted based on 160 participants with data from the fecal SCFA. The same covariates as described above were included in the mediation analysis.

Statistical analyses were conducted using SAS (version 9.4, SAS Institute, Cary, NC, USA) and R (version 4.1.2, R Foundation for Statistical Computing). The “mediation” and “pwrss” packages in R were used for the mediation analysis as well as for sample size and statistical power calculations. A *P* value of less than 0.05 was deemed statistically significant.

### Ethics approval and consent to participate

Approval of the research protocol by an Institutional Reviewer Board: This study was approved by the Research Ethics Review Committee of the National Institutes of Biomedical Innovation, Health and Nutrition (No. Kenei 3-10 and Kenei 102-04).

### Informed consent

The procedures of the study and the risks associated with participation were explained to the subjects, and written informed consent was obtained from all participants.

### Supplementary Information

Supplementary Information.

## Data Availability

The datasets analyzed during the current study are available from the corresponding author on reasonable request.
