# Supplementary material for: Association between physical activity and the prevalence of tumorigenic bacteria in the gut microbiota of Japanese adults: a cross-sectional study
Source: Sci Rep. 2023 Nov 27;13:20841. doi: 10.1038/s41598-023-47442-9 (PMC10682492; doi:10.1038/s41598-023-47442-9)
Supplement: Supplementary file 1 — Supplementary Information. [file 41598_2023_47442_MOESM1_ESM.docx]

**Supporting information:**

**Supplementary Table 1**. Demographic characteristics based on tertile of LPA

**Supplementary Table 2**. Demographic characteristics based on tertile of MVPA

**Supplementary Table 3**. Demographic characteristics based on tertile of time spent inactive

**Supplementary Table 4**. Demographic characteristics based on tertile of PAL

**Supplementary Table 5**. Demographic characteristics based on tertile of step-count

**Supplementary Table 6**. Mediation analysis of the relationship between LPA on *pks^+^ E. coli* via short-chain fatty acids (n = 160)

**Supplementary Table 7**. Mediation analysis of the relationship between MVPA on *pks^+^ E. coli* via short-chain fatty acids (n = 160)

**Supplementary Table 8**. Mediation analysis of the relationship between the time spent inactive on *pks^+^ E. coli* via short-chain fatty acids (n = 160)

**Supplementary Table 9**. Mediation analysis of the relationship between PAL on *pks^+^ E. coli* via short-chain fatty acids (n = 160)

**Supplementary Table 10**. Mediation analysis of the relationship between the step-count on *pks^+^ E. coli* via short-chain fatty acids (n = 160)

**Supplementary Figure 1**. Restricted cubic spline curves showing the dose-response relationship between the prevalence of *pks^+^ Escherichia coli* and each physical activity variable by sex. (a) Light intensity physical activity (LPA), (b) moderate-to-vigorous physical activity (MVPA), (c) time spent inactive, (d) physical activity level (PAL), (e) step-count. The solid lines represent odds ratios (ORs) and the band areas represent 95% confidence intervals. The red and blue colors indicates female and male, respectively. The Y-axis is shown on the logarithmic axis. All dose-response relationships were adjusted for age, sex, body mass index, drinking, smoking, a family history of cancer, energy intake, and green tea intake.

**Supplementary Figure 2**. Restricted cubic spline curves showing the dose-response relationship between the prevalence of *pks^+^ Escherichia coli* and each physical activity variable by age group (60+ and <60). (a) Light intensity physical activity (LPA), (b) moderate-to-vigorous physical activity (MVPA), (c) time spent inactive, (d) physical activity level (PAL), (e) step-count. The solid lines represent odds ratios (ORs) and the band areas represent 95% confidence intervals. The red and blue colors indicates female and male, respectively. The Y-axis is shown on the logarithmic axis. All dose-response relationships were adjusted for age, sex, body mass index, drinking, smoking, a family history of cancer, energy intake, and green tea intake.

| Supplementary table 1. Demographic characteristics based on tertile of LPA | | | | |
| --- | --- | --- | --- | --- |
|  | Tertile of LPA |  |  |  |
| Characteristics | T1, n = 74 | T2, n = 74 | T3, n = 74 | *P* for trend |
| *pks^+^ E. coli*, n [%] | 26 [35.1%] | 19 [25.7%] | 14 [18.9%] | 0.026 |
| Age, y | 55.4 (13.3) | 58.8 (12.0) | 62.1 (10.8) | 0.001 |
| Female, n [%] | 35 [47.3%] | 59 [79.7%] | 70 [94.6%] | <0.001 |
| Height, cm | 164.2 (8.0) | 159.7 (7.7) | 157.0 (7.0) | <0.001 |
| Weight, kg | 61.5 (9.3) | 58.2 (8.8) | 53.7 (7.7) | <0.001 |
| BMI, kg/m^2^ | 22.8 (2.5) | 22.8 (3.0) | 21.8 (2.6) | 0.027 |
| Family history of cancer, n [%] | 40 [54.1%] | 39 [52.7%] | 46 [62.2%] | 0.321 |
| LPA, min/day | 240 (40) | 356 (26) | 465 (45) | <0.001 |
| MVPA, min/day | 64 (31) | 59 (25) | 69 (31) | 0.314 |
| Inactivity, min/day | 1137 (55) | 1025 (37) | 907 (54) | <0.001 |
| PAL | 1.63 (0.12) | 1.75 (0.09) | 1.89 (0.11) | <0.001 |
| Step-count, steps/day | 9,665 (3,429) | 9,298 (2,807) | 9,940 (3,526) | 0.610 |
| Energy intake, kcal/day | 1,780 (484) | 1,726 (427) | 1,679 (437) | 0.171 |
| Green tea intake, g/1000kcal/day | 126 (125) | 120 (140) | 162 (140) | 0.106 |
| Alcohol drinker, n [%] | 39 [52.7%] | 32 [43.2%] | 44 [59.5%] | 0.412 |
| Smoking, n [%] |  |  |  | 0.044 |
| current | 3 [4.1%] | 2 [2.7%] | 3 [4.1%] |  |
| former | 23 [31.1%] | 17 [23.0%] | 10 [13.5%] |  |
| Never | 48 [64.9%] | 55 [74.3%] | 61 [82.4%] |  |
| T, tertile; *pks^+^ E. coli*, polyketide synthase *Escherichia coli* positive; BMI, body mass index; LPA, light-intensity physical activity; MVPA, moderate-to-vigorous physical activity; PAL, physical activity level. Continuous: mean (SD), tested with a t-test; categorical: n [%], tested with a chi-square test. | | | | |

| Supplementary table 2. Demographic characteristics based on tertile of MVPA | | | | |
| --- | --- | --- | --- | --- |
|  | Tertile of MVPA |  |  |  |
| Characteristics | T1, n = 74 | T2, n = 74 | T3, n = 74 | *P* for trend |
| *pks^+^ E. coli*, n [%] | 21 [28.4%] | 19 [25.7%] | 19 [25.7%] | 0.710 |
| Age, y | 61.4 (12.2) | 58.7 (13.1) | 56.1 (11.3) | 0.008 |
| Female, n [%] | 57 [77.0%] | 55 [74.3%] | 52 [70.3%] | 0.351 |
| Height, cm | 159.3 (8.2) | 160.9 (7.3) | 160.7 (8.7) | 0.297 |
| Weight, kg | 57.7 (9.1) | 58.0 (8.7) | 57.8 (9.9) | 0.924 |
| BMI, kg/m^2^ | 22.7 (3.1) | 22.4 (2.8) | 22.3 (2.3) | 0.317 |
| Family history of cancer, n [%] | 39 [52.7%] | 44 [59.5%] | 42 [56.8%] | 0.620 |
| LPA, min/day | 345 (96) | 346 (105) | 370 (97) | 0.135 |
| MVPA, min/day | 37 (9) | 59 (6) | 95 (26) | <0.001 |
| Inactivity, min/day | 1,058 (97) | 1,035 (104) | 975 (101) | <0.001 |
| PAL | 1.66 (0.11) | 1.73 (0.11) | 1.87 (0.14) | <0.001 |
| Step-count, steps/day | 7,038 (1,620) | 9,278 (1,580) | 12,587 (3,371) | <0.001 |
| Energy intake, kcal/day | 1,681 (435) | 1,784 (487) | 1,720 (426) | 0.597 |
| Green tea intake, g/1000kcal/day | 141 (142) | 150 (138) | 116 (127) | 0.270 |
| Alcohol drinker, n [%] | 38 [51.4%] | 43 [58.1%] | 34 [45.9%] | 0.512 |
| Smoking, n [%] |  |  |  | 0.089 |
| current | 5 [6.8%] | 3 [4.1%] | 0 [0.0%] |  |
| former | 16 [21.6%] | 19 [25.7%] | 15 [20.3%] |  |
| never | 53 [71.6%] | 52 [70.3%] | 59 [79.7%] |  |
| T, tertile; *pks^+^ E. coli*, polyketide synthase *Escherichia coli* positive; BMI, body mass index; LPA, light-intensity physical activity; MVPA, moderate-to-vigorous physical activity; PAL, physical activity level. Continuous: mean (SD), tested with a t-test; categorical: n [%], tested with a chi-square test. | | | | |

| Supplementary table 3. Demographic characteristics based on tertile of time spent inactive | | | | |
| --- | --- | --- | --- | --- |
|  | Tertile of time spent inactive | | |  |
| Characteristics | T1, n = 74 | T2, n = 74 | T3, n = 74 | *P* for trend |
| *pks^+^ E. coli*, n [%] | 18 [24.3%] | 14 [18.9%] | 27 [36.5%] | 0.095 |
| Age, y | 60.7 (10.6) | 58.2 (13.2) | 57.3 (12.9) | 0.088 |
| Female, n [%] | 68 [91.9%] | 57 [77.0%] | 39 [52.7%] | <0.001 |
| Height, cm | 157.6 (6.8) | 160.5 (8.7) | 162.9 (7.9) | <0.001 |
| Weight, kg | 54.3 (7.4) | 58.4 (10.1) | 60.7 (8.8) | <0.001 |
| BMI, kg/m^2^ | 21.9 (2.4) | 22.6 (3.0) | 22.8 (2.7) | 0.029 |
| Family history of cancer, n [%] | 44 [59.5%] | 39 [52.7%] | 42 [56.8%] | 0.741 |
| LPA, min/day | 460 (52) | 355 (38) | 245 (48) | <0.001 |
| MVPA, min/day | 76 (34) | 61 (27) | 54 (21) | <0.001 |
| Inactivity, min/day | 903 (50) | 1,023 (28) | 1,141 (46) | <0.001 |
| PAL | 1.91 (0.10) | 1.75 (0.07) | 1.60 (0.09) | <0.001 |
| Step-count, steps/day | 10,574 (3,727) | 9,653 (3,192) | 8,676 (2,525) | <0.001 |
| Energy intake, kcal/day | 1,699 (440) | 1,717 (427) | 1,768 (484) | 0.352 |
| Green tea intake, g/1000kcal/day | 156 (139) | 119 (136) | 132 (131) | 0.272 |
| Alcohol drinker, n [%] | 43 [58.1%] | 33 [44.6%] | 39 [52.7%] | 0.512 |
| Smoking, n [%] |  |  |  | 0.089 |
| current | 3 [4.1%] | 2 [2.7%] | 3 [4.1%] |  |
| former | 11 [14.9%] | 17 [23.0%] | 22 [29.7%] |  |
| never | 60 [81.1%] | 55 [74.3%] | 49 [66.2%] |  |
| T, tertile; *pks^+^ E. coli*, polyketide synthase *Escherichia coli* positive; BMI, body mass index; LPA, light-intensity physical activity; MVPA, moderate-to-vigorous physical activity; PAL, physical activity level. Continuous: mean (SD), tested with a t-test; categorical: n [%], tested with a chi-square test. | | | | |

| Supplementary table 4. Demographic characteristics based on tertile of PAL | | | | |
| --- | --- | --- | --- | --- |
|  | Tertile of PAL |  |  |  |
| Characteristics | T1, n = 74 | T2, n = 74 | T3, n = 74 | *P* for trend |
| *pks^+^ E. coli*, n [%] | 26 [35.1%] | 16 [21.6%] | 17 [23.0%] | 0.095 |
| Age, y | 58.2 (13.7) | 59.1 (11.7) | 58.9 (11.6) | 0.720 |
| Female, n [%] | 42 [56.8%] | 61 [82.4%] | 61 [82.4%] | <0.001 |
| Height, cm | 161.7 (8.5) | 160.7 (7.5) | 158.5 (8.0) | 0.010 |
| Weight, kg | 59.8 (8.9) | 57.7 (8.5) | 56.0 (9.8) | 0.112 |
| BMI, kg/m^2^ | 22.9 (2.8) | 22.3 (2.8) | 22.2 (2.6) | 0.018 |
| Family history of cancer, n [%] | 38 [51.4%] | 42 [56.8%] | 45 [60.8%] | 0.247 |
| LPA, min/day | 258 (64) | 362 (58) | 441 (75) | <0.001 |
| MVPA, min/day | 47 (18) | 59 (20) | 85 (33) | <0.001 |
| Inactivity, min/day | 1,135 (56) | 1,019 (45) | 914 (65) | <0.001 |
| PAL | 1.59 (0.07) | 1.75 (0.04) | 1.92 (0.09) | <0.001 |
| Step-count, steps/day | 7,976 (2,074) | 9,228 (2,378) | 11,699 (3,879) | <0.001 |
| Energy intake, kcal/day | 1,782 (475) | 1,654 (440) | 1,749 (430) | 0.655 |
| Green tea intake, g/1000kcal/day | 130 (123) | 136 (153) | 141 (132) | 0.622 |
| Alcohol drinker, n [%] | 40 [54.1%] | 34 [45.9%] | 41 [55.4%] | 0.870 |
| Smoking, n [%] |  |  |  | 0.122 |
| current | 3 [4.1%] | 3 [4.1%] | 2 [2.7%] |  |
| former | 22 [29.7%] | 14 [18.9%] | 14 [18.9%] |  |
| never | 49 [66.2%] | 57 [77.0%] | 58 [78.4%] |  |
| T, tertile; *pks^+^ E. coli*, polyketide synthase *Escherichia coli* positive; BMI, body mass index; LPA, light-intensity physical activity; MVPA, moderate-to-vigorous physical activity; PAL, physical activity level. Continuous: mean (SD), tested with a t-test; categorical: n [%], tested with a chi-square test. | | | | |

| Supplementary table 5. Demographic characteristics based on tertile of step-count | | | | |
| --- | --- | --- | --- | --- |
|  | Tertile of step-count | | |  |
| Characteristics | T1, n = 74 | T2, n = 74 | T3, n = 74 | *P* for trend |
| *pks^+^ E. coli*, n [%] | 21 [28.4%] | 16 [21.6%] | 22 [29.7%] | 0.853 |
| Age, y | 61.5 (11.9) | 58.8 (12.8) | 55.9 (11.7) | 0.005 |
| Female, n [%] | 60 [81.1%] | 57 [77.0%] | 47 [63.5%] | 0.015 |
| Height, cm | 159.2 (7.5) | 160.1 (7.7) | 161.6 (8.9) | 0.077 |
| Weight, kg | 57.1 (8.1) | 57.9 (9.0) | 58.5 (10.3) | 0.358 |
| BMI, kg/m^2^ | 22.5 (2.7) | 22.6 (3.2) | 22.3 (2.3) | 0.559 |
| Family history of cancer, n [%] | 40 [54.1%] | 42 [56.8%] | 43 [58.1%] | 0.620 |
| LPA, min/day | 350 (99) | 341 (100) | 370 (98) | 0.233 |
| MVPA, min/day | 41 (13) | 58 (14) | 92 (28) | <0.001 |
| Inactivity, min/day | 1,049 (103) | 1,041 (100) | 978 (102) | <0.001 |
| PAL | 1.67 (0.13) | 1.72 (0.12) | 1.87 (0.14) | <0.001 |
| Step-count, steps/day | 6,588 (1,185) | 9,260 (663) | 13,056 (3,003) | <0.001 |
| Energy intake, kcal/day | 1,646 (403) | 1,783 (483) | 1,756 (455) | 0.135 |
| Green tea intake, g/1000kcal/day | 150 (149) | 149 (137) | 108 (117) | 0.056 |
| Alcohol drinker, n [%] | 38 [51.4%] | 45 [60.8%] | 32 [43.2%] | 0.325 |
| Smoking, n [%] |  |  |  | 0.216 |
| current | 4 [5.4%] | 4 [5.4%] | 0 [0.0%] |  |
| Former | 18 [24.3%] | 14 [18.9%] | 18 [24.3%] |  |
| Never | 52 [70.3%] | 56 [75.7%] | 56 [75.7%] |  |
| T, tertile; *pks^+^ E. coli*, polyketide synthase *Escherichia coli* positive; BMI, body mass index; LPA, light-intensity physical activity; MVPA, moderate-to-vigorous physical activity; PAL, physical activity level. Continuous: mean (SD), tested with a t-test; categorical: n [%], tested with a chi-square test. | | | | |

Supplementary Table 6. Mediation analysis of the relationship between LPA on pks+ E. coli via short-chain fatty acids (n = 160)

| Measure | Estimate | 95% CI Lower | 95% CI Upper | p value |
| --- | --- | --- | --- | --- |
| Total Effect | −0.046 | −0.15 | 0.06 | 0.39 |
| ACME | 0.002 | −0.01 | 0.02 | 0.71 |
| ADE | −0.048 | −0.15 | 0.06 | 0.39 |
| Prop. Mediated | −0.008 | −0.98 | 0.87 | 0.86 |

LPA, light-intensity physical activity; ACME, average causal mediation effect; ADE, average direct effect; Prop. Mediated; proportions mediated.

Supplementary Table 7. Mediation analysis of the relationship between MVPA on *pks^+^ E. coli* via short-chain fatty acids (n = 160)

| Measure | Estimate | 95% CI Lower | 95% CI Upper | p value |
| --- | --- | --- | --- | --- |
| Total Effect | 0.001 | −0.09 | 0.08 | 0.92 |
| ACME | 0.002 | −0.01 | 0.02 | 0.69 |
| ADE | −0.001 | −0.09 | 0.08 | 0.97 |
| Prop. Mediated | 0.002 | −1.46 | 1.13 | 0.98 |

MVPA, moderate-to-vigorous physical activity; ACME, average causal mediation effect; ADE, average direct effect; Prop. Mediated; proportions mediated.

Supplementary Table 8. Mediation analysis of the relationship between the time spent inactive on pks+ E. coli via short-chain fatty acids (n = 160)

| Measure | Estimate | 95% CI Lower | 95% CI Upper | p value |
| --- | --- | --- | --- | --- |
| Total Effect | 0.025 | −0.071 | 0.11 | 0.57 |
| ACME | −0.003 | −0.021 | 0.01 | 0.61 |
| ADE | 0.028 | −0.069 | 0.11 | 0.53 |
| Prop. Mediated | −0.012 | −1.147 | 1.16 | 0.84 |

ACME, average causal mediation effect; ADE, average direct effect; Prop. Mediated; proportions mediated.

Supplementary Table 9. Mediation analysis of the relationship between PAL on pks+ E. coli via short-chain fatty acids (n = 160)

| Measure | Estimate | 95% CI Lower | 95% CI Upper | p value |
| --- | --- | --- | --- | --- |
| Total Effect | −0.041 | −0.15 | 0.05 | 0.38 |
| ACME | 0.000 | −0.01 | 0.01 | 0.98 |
| ADE | −0.041 | −0.15 | 0.05 | 0.39 |
| Prop. Mediated | 0.000 | −0.79 | 0.59 | 0.99 |

PAL, physical activity level; ACME, average causal mediation effect; ADE, average direct effect; Prop. Mediated; proportions mediated.

Supplementary Table 10. Mediation analysis of the relationship between the step-count on pks+ E. coli via short-chain fatty acids (n = 160)

| Measure | Estimate | 95% CI Lower | 95% CI Upper | p value |
| --- | --- | --- | --- | --- |
| Total Effect | 0.014 | −0.08 | 0.10 | 0.72 |
| ACME | 0.000 | −0.01 | 0.01 | 1.00 |
| ADE | 0.015 | −0.08 | 0.10 | 0.72 |
| Prop. Mediated | 0.002 | −1.01 | 0.95 | 0.96 |

ACME, average causal mediation effect; ADE, average direct effect; Prop. Mediated; proportions mediated.

**
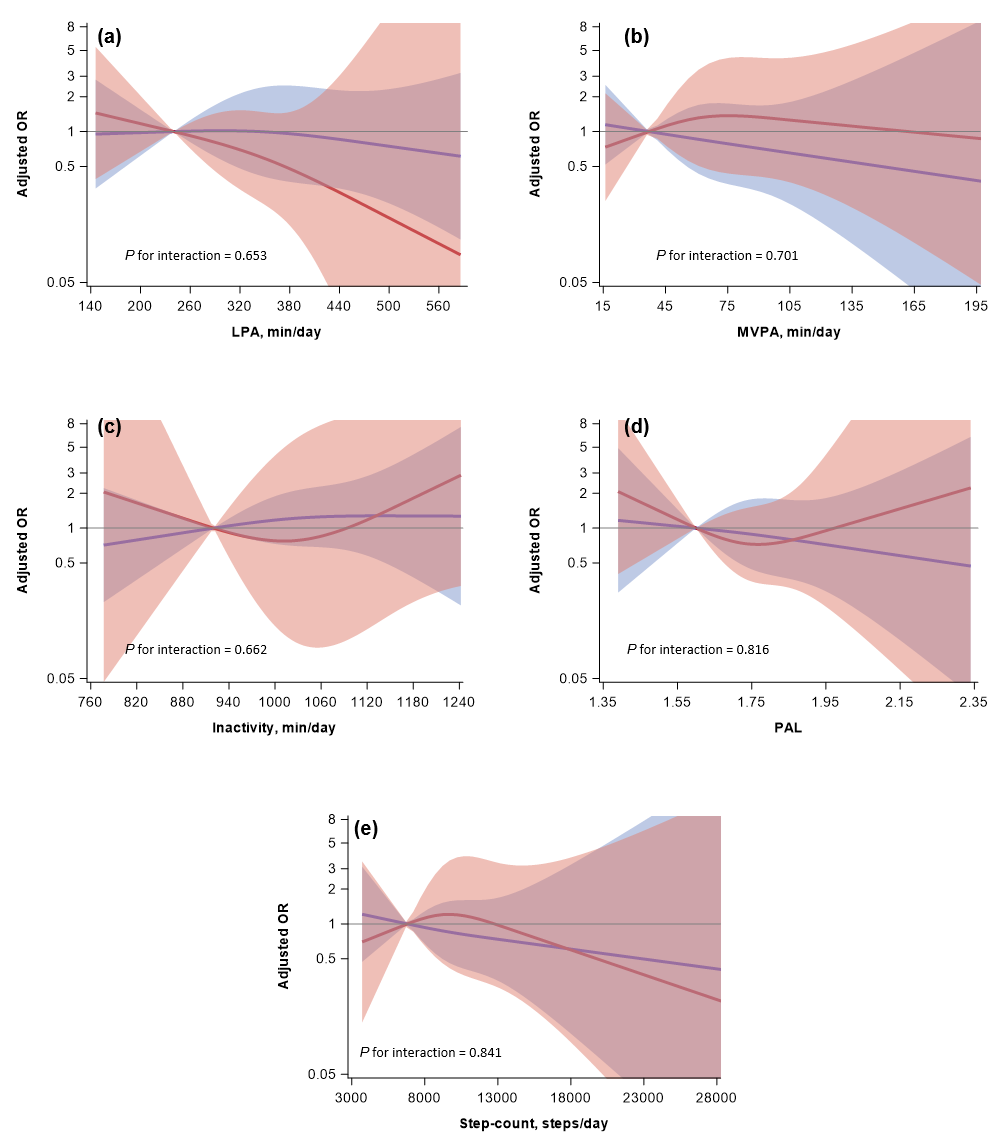
**

**Supplementary Figure 1**. Restricted cubic spline curves showing the dose-response relationship between the prevalence of *pks^+^ Escherichia coli* and each physical activity variable by sex. (a) Light intensity physical activity (LPA), (b) moderate-to-vigorous physical activity (MVPA), (c) time spent inactive, (d) physical activity level (PAL), (e) step-count. The solid lines represent odds ratios (ORs) and the band areas represent 95% confidence intervals. The red and blue colors indicates female and male, respectively. The Y-axis is shown on the logarithmic axis. All dose-response relationships were adjusted for age, sex, body mass index, drinking, smoking, a family history of cancer, energy intake, and green tea intake.


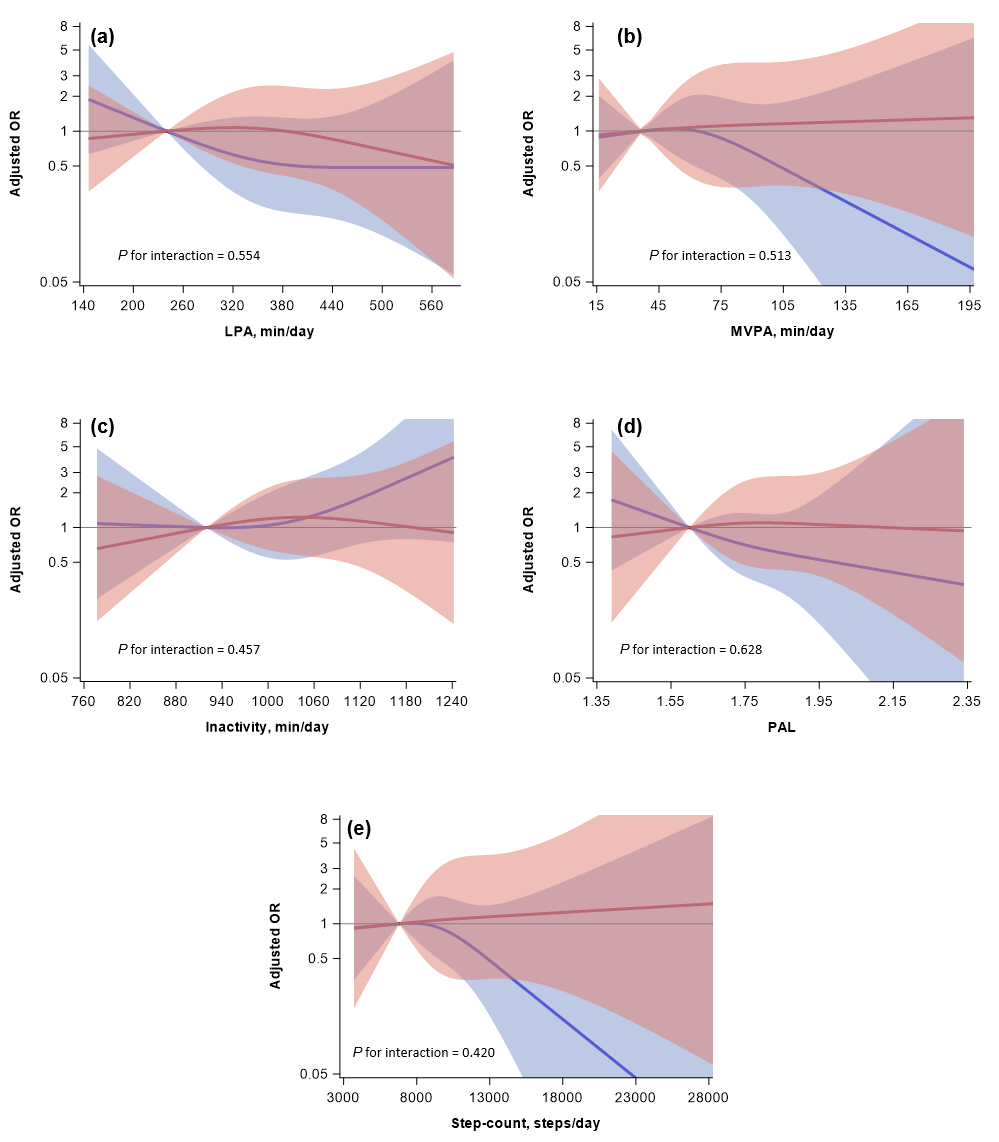


**Supplementary Figure 2**. Restricted cubic spline curves showing the dose-response relationship between the prevalence of *pks^+^ Escherichia coli* and each physical activity variable by age group (60+ and <60). (a) Light intensity physical activity (LPA), (b) moderate-to-vigorous physical activity (MVPA), (c) time spent inactive, (d) physical activity level (PAL), (e) step-count. The solid lines represent odds ratios (ORs) and the band areas represent 95% confidence intervals. The red and blue colors indicates <60 and 60+, respectively. The Y-axis is shown on the logarithmic axis. All dose-response relationships were adjusted for age, sex, body mass index, drinking, smoking, a family history of cancer, energy intake, and green tea intake.
